# The complete nucleotide sequence of the mitochondrial genome of *Epicauta aptera* Kaszab

**DOI:** 10.1080/23802359.2016.1192500

**Published:** 2016-07-10

**Authors:** Hang Jie, Meiyan Lei, Pinming Li, Xiaolan Feng, Dejun Zeng, Guijun Zhao, Jibin Zhu, Chenglu Zhang, Mi Yu, Ya Huang, Qiang Chen

**Affiliations:** Laboratory of Medicinal Animal, Chongqing Institute of Medicinal Plant Cultivation, Chongqing, P. R. China

**Keywords:** Complete mitochondrial genome, *Epicauta aptera* Kaszab, sequencing

## Abstract

*Epicauta aptera* Kaszab *(E. aptera)*, an endangered species used to extract cantharidin in Chinese traditional medicine, has a limited geographical distribution in the southwest China. The complete mitochondrial genome of *E. aptera* is 15 645bp which contains an A + T rich region and 37 genes, including 13 protein-coding genes, 22 tRNA genes and two rRNA genes (GenBank accession No. KX023302). All protein-coding genes are initiated by ATN start codon. Moreover, the largest non-coding A + T-rich region with a length of 1039 bp is at the end of 12S rRNA. It is the first report involved in the complete mitochondrial genome of *E. aptera*.

*E. aptera*, belonging to the Meloidae, used to extract cantharidin in Chinese traditional medicine, is widely distributed in southwestern Japan (Mochida 2003), but has a limited geographical distribution in the southwest China (Guo 2014). We sequenced the complete mitochondrial genome of *E. aptera*, by next-generation sequencing (NGS) techniques strategy according to method of Xie et al (2015). The sample was obtained from Nanchuan District, Chongqing, China (N: 29° 08’ 00.21’’, E: 107° 12’ 03.42’’). When the living sample was collected, viscera were removed to prevent pollution and the sample was returned to the lab and stored at −80 °C and labeled with an accession number EACQ003. The total genomic DNA was extracted from abdomen of *E. aptera* by salting-out procedure (Howe et al. 1997). Mitochondrial DNA of *E. aptera* was sequenced in Shanghai Sangon Biotech limited company (Xiangmin Road, Shongjiang District, Shanghai, P.R. China).

The mitochondrial genome organization and gene arrangement pattern were identical to the typical insect mitochondrial genome (Wei et al. 2015). The complete mitochondrial DNA of *E. aptera* is 15 645 bp in length (GenBank accession No. KX023302). It contains an A + T rich region (D-loop) and 37 genes, including 13 protein-coding genes (ATP6, ATP8, COX I-III, ND1, 2, 3, 4, 4L, 5, 6 and Cytb), two ribosomal RNA genes (12S rRNA and 16S rRNA), 22 transfer RNA (tRNA) genes. Except for ND1, 4, 4L, 5 encoded on the L-strand, most of the protein-coding genes are encoded on the H-strand.

The overall nucleotides composition of the *E. aptera* mitochondrial DNA is 35.57% A, 31.90% T, 12.46% G and 20.06% C, with an AT bias of 67.47%. There are six overlapping region and 21 intergenic spacer regions found in the mitochondrial genome. All protein-coding genes are initiated by ATN start codon, among which, only two protein-coding genes, including ND4 and COX3 gene, are initiate with the regular codon ATG. Nine different patterns of termination codons are found, including TTC for ND2 gene, GAA for COX1 and ATP6 gene, ATA for COX2 and Cyt b gene, CTA for ATP8 gene, AGA for COX3 gene, TGA for ND3 gene, TTA for ND4, 4L, 5 gene, CCA for ND6 gene, AGC for ND1 gene. The mitochondrial genome of *E. aptera* contains 22 transfer RNA genes, ranging from 57 to 69 bp, has a typical cloverleaf structure. The large mitochondrial rRNA subunit (16S rRNA) is 1 051 bp, while the small subunit (12S rRNA) has a total length of 765 bp. The largest non-coding A + T-rich region (D-loop) is 1039 bp long, which is shorter than the 16S rRNA (1051 bp). The A + T content of D-loop is up to 77.28%, which was believed to participate in the regulation of transcription and control of DNA replication (Zhang & Hewitt 1997). A NJ tree of 18 insect and 2 outgroup based on mitogenome was constructed by MEGA6 (Figure 1). The NJ bootstrap for 500 replicates was indicated in each node. The evolutionary position of *E. aptera* was showed in the NJ tree.

**Figure 1.**
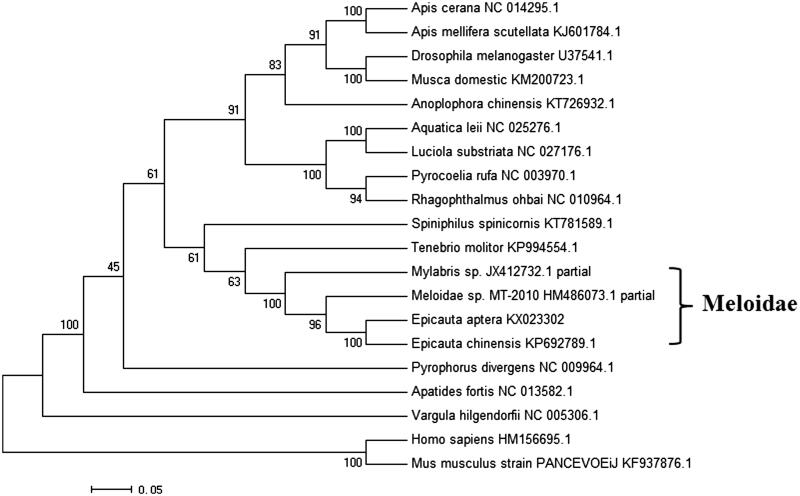
Evolutionary relationships of 18 insect and 2 outgroup species. The mitochondrial genome of Meloidae has been marked.
